# Editorial: Bacteriocins and other ribosomally synthesised and post-translationally modified peptides (RiPPs) as modulators of the microbiome

**DOI:** 10.3389/fmicb.2024.1463909

**Published:** 2024-08-23

**Authors:** Harsh Mathur, Des Field, Caitriona Guinane, Maire Begley, Mathew Upton, Hilario C. Mantovani, Paul D. Cotter

**Affiliations:** ^1^Teagasc Food Research Centre, Moorepark, Cork, Ireland; ^2^School of Microbiology, University College Cork, Cork, Ireland; ^3^School of Microbiology, APC Microbiome Ireland, University College Cork, Cork, Ireland; ^4^Biological Sciences Department, Munster Technological University, Cork, Ireland; ^5^School of Biomedical Sciences, University of Plymouth, Plymouth, United Kingdom; ^6^Animal and Dairy Sciences Department, University of Wisconsin-Madison, Madison, WI, United States; ^7^VistaMilk, Cork, Ireland

**Keywords:** bacteriocins, peptides, microbiome, modulating, antimicrobials

This Research Topic (RT) entitled “*Bacteriocins and Ribosomally Synthesised Post-Translationally modified Peptides (RiPPs) as modulators of the microbiome*” focussed on various aspects of these peptides. It includes seven research articles that cover a variety of related areas, including testing the impact of a range of bacteriocins on a simplified human intestinal microbiota, the investigation of a regulator involved in bacteriocin production, elucidation of the mechanism of action of a lantibiotic, optimization of the heterologous production of circular bacteriocins, and utilization of *in silico* tools to identify novel bacteriocins, amongst other topics. The RT also includes a timely review article relating to the use of bacteriocin like inhibitory substances (BLIS) to strategically shape the oral microbiota. Here we briefly describe these studies and the insights they provide with respect to the ability of RiPPs to modulate microbiomes.

One study relates to the use of several bacteriocin-producing (Bac+) strains to evaluate the impact these antimicrobials have on specific bacterial populations in a simplified human intestinal microbiota (SIHUMI) (Ríos Colombo et al.). The SIHUMI consortium is composed of 7 strains from the species *Escherichia coli, Enterococcus faecalis, Lactiplantibacillus plantarum, Faecalibacterium prausnitzii, Bifidobacterium longum, Phocaeicola vulgatus*, and *Ruminococcus gnavus*. The authors tested several Bac+ strains as well as their isogenic non-bacteriocin producing counterparts (Bac–), which were used as negative controls. Amongst the bacteriocin producing strains were producers of the lantibiotics nisin A and lacticin 3147, as well as producers of class II, non-modified, bacteriocins such as pediocin PA-1. Quantitative PCR (qPCR) was used to quantify the levels of the 7 SIHUMI strains throughout these experiments. It was found that the composition of the SIHUMI community was altered in various ways with some unexpected off-target effects observed. The authors hypothesized that such off-target effects involved antagonistic inter-species interactions within the consortium and suggested that such effects might be harnessed deliberately to manipulate and strategically shape specific complex communities within certain microbiomes, including the human gut microbiome.

McAnulty et al. focused on the investigation of BlpC, a quorum sensing peptide, and its role in regulating gene transcription outside of its associated gene cluster and discussing how it has an impact on the growth of *Streptococcus thermophilus* (McAnulty et al.). Somewhat similar to the aforementioned paper by Ríos Colombo et al., this study relied heavily on the use of qPCR as the main technique. In several *Strep. thermophilus* strains, BlpC regulates the transcription of bacteriocin-associated genes in a cell density dependent manner. However, in certain strains such as *Strep. thermophilus* ST106, BlpC needs to be added exogenously because the quorum sensing system for bacteriocin production in this strain does not always work efficiently. In contrast, the production of the bacteriocin thermophilin 110 in *Strep. thermophilus* strain B59671 takes place naturally without the BlpC regulator. In this study, the authors evaluated global gene expression in strain ST106 with or without synthetic BlpC, and in strain B59671 in the presence of BlpC to ascertain if the expression of genes outside of the *blp* cluster was affected or not. Interestingly, it was found that in strain ST106, BlpC had an impact on the expression of the transcription regulators such as a putative YtrA-subfamily transcriptional repressor, which resides outside the *blp* gene cluster. BlpC diminished the production of a separate bacteriocin, thermophilin 13, while inducing the YtrA-subfamily transcriptional repressor in strain B59671. The authors were also able to demonstrate that thermophilin 110 was responsible for the broad-spectrum antimicrobial activity of strain B59671 whereas thermophilin 13 is involved in inhibiting intra-species growth. The induction or production of BlpC suppressed the growth of strains ST106 and B59671 in the study, thereby confirming its role as a quorum sensing mediated regulator and confirming the selective pressure not to produce bacteriocins unless required. The implications of this study are that the authors were able to demonstrate that BlpC has an impact on the regulation of expression of additional genes outside the bacteriocin cluster, which has the potential to be strategically manipulated with a view to optimizing bacteriocin production for several applications pertaining to human and animal health and in agriculture.

A follow-up study by the same research group reported the characterization of the broad-spectrum antimicrobial peptide BlpU produced by *Strep. thermophilus* B59671 (Renye et al.). The associated *blp* gene cluster was analyzed and two genes *blpU* and *blpK* were identified as putative bacteriocin-encoding genes. An abolition of antimicrobial activity against *Strep. thermophilus* ST113 or *Pediococcus acidilactici* indicator strains was not seen when the *blpK* gene was deleted from the B59671 chromosome. While deletion of the *blpU* gene was not possible, chemical synthesis of the BlpU peptide or overexpression of the mature BlpU peptide in *Escherichia coli* inhibited *Strep. mutans, P. acidilactici, Listeria innocua*, and *Strep. thermophilus* target strains. This indicated that modification of the BlpU peptide is not required for its antimicrobial activity. Deletion of the *blpC* gene in B59671 inhibited BlpU and BlpK expression and as a consequence of the resultant disruption of thermophilin 13 production, prevented the growth of *Bacillus cereus* and *Strep. thermophilus* indicator strains, thus highlighting the important role of *blpC* in overall antimicrobial activity. Overall, the study verified that the BlpU peptide can serve as an independent antimicrobial peptide while additional studies are warranted to ascertain whether BlpK can function as a broad-spectrum antimicrobial independently or if it requires other peptide(s) to exert antimicrobial activity.

Another intriguing study by Wu et al., provided insights into the mode of action of the lantibiotic epilacin 15X (Wu et al.). This lantibiotic is produced by *Staphylococcus epidermidis* 15X154 and possesses potent antimicrobial activity in the low micromolar range against certain pathogenic Gram positive targets. In the study, the authors showed that epilacin 15X causes membrane disruption in sensitive *Staphylococcus carnosus* and *Bacillus subtilis* strains and has bactericidal activity. The authors demonstrated that the conserved cationic residues of epilcancin 15X and dehydroamino acids are crucial for bioactivity while the N-terminal lactyl group is less important, and changes in these N-terminal regions have less of an impact on bioactivity. It was also shown that epilancin 15X suppresses DNA replication, transcription, RNA translation and fatty acid synthesis but does not affect cell wall biosynthesis. Epilancin 15X disrupts the membranes of liposomes, which consist of anionic membrane lipids, in a lipid II independent manner. Lipid II is a peptidoglycan and a key component involved in bacterial cell wall synthesis and is the target of several antimicrobials, including several classes of antibiotics as well as bacteriocins. In terms of affecting regulators, epilancin 15X induced a LiasRS response regulator in *B. subtilis* but did not upregulate VraRS in *S. carnosus*. Finally, exposure to epilcancin 15X results in an aggregation phenotype in *B. subtilis* and *S. carnosus*, as observed by microscopy. Thus, the authors provided comprehensive insights into the mechanism of action of epilcancin 15X against selective targets.

This RT also contains a description of optimized heterologous expression of the bacteriocin garvicin Q in *Corynebacterium glutamicum* (Desiderato et al.). The authors noted that the *C. glutamicum* host secreted approximately 7 mg/L of GarQ in initial fermentations. One of the limiting factors was the fact that the positively charged peptide was electrostatically attracted to the anionic envelope of producer strains and thus adsorbed to the cell envelope, reducing the concentrations of the peptide available in cell-free supernatants. While the authors demonstrated that factors such as adding Tween 80 and CaCl_2_, and growing *C. glutamicum* in minimal media, decreased the adsorption of GarQ to the *C. glutamicum* cell envelope, and increased production somewhat, further optimization to increase yields was necessary. The authors also found that the protease HtrA, produced by the host, also resulted in decreased titres. Therefore, using a HtrA-deficient *C. glutamicum* K9 heterologous host ameliorated GarQ titers to approximately 40 mg/L, while introducing environmental parameters of low aeration further enhanced GarQ titers to approximately 100 mg/L, a level which was comparable to the titers of the native producing strain *Lactococcus petauri* B1726. In addition to optimization of the conditions for heterologous production and obtaining optimal yields, the authors identified synthetic variants of GarQ that demonstrated enhanced antimicrobial activity against *Listeria monocytogenes* and *Lactococcus lactis* as a consequence of substituting methionine in residue 5 to phenylalanine (GarQ M5F). Overall, this study was particularly important in providing insights into optimizing heterologous expression of garvicin Q and the work has potential implications for groups developing such systems for scaling up production.

In contrast to the other studies published as part of this RT, the paper by Peña et al. focused on circular bacteriocins. These circular bacteriocins undergo a head-to-tail circularization after biosynthesis, and this circularization confers temperature and pH stability, as well as enhanced resistance to proteolytic cleavage, thereby contributing to their potential applications. While several operons which could potentially code for circular bacteriocins have been found through genome mining studies, there has been a relative dearth of circular bacteriocins that have been fully characterized in the laboratory. This can be attributed to the fact that their synthesis, including through heterologous expression, is complex due to a requirement for several genes involved in synthesis and circularization. In this study, the authors optimized a synthetic biology strategy for the *in vivo* and *in vitro* production of garvicin ML (GarML) by utilizing a split-intein mediated ligation (SIML) approach. Crucially, the authors showed that the expression of just one gene is sufficient to produce a protein that circularizes to an active peptide form, after intein splicing. This intein splicing approach was crucial in ensuring that the resultant peptide possesses the same amino acid sequence and molecular mass as the native GarML peptide. These findings and the utilization of this SIML tool could have tremendous biotechnological potential with a view to producing, bioengineering and enhancing the antimicrobial potential of several circular bacteriocins.

This RT also included an *in silico* study by Fernandez-Cantos et al. focusing on mining for RiPP biosynthetic gene clusters (BGCs), and clusters with the potential to encode class I bacteriocins in particular, from within the Bacteroidales order (Fernandez-Cantos et al.). This order of bacteria is a major component of the human microbiota with several key functions including modulation of the host immune system, involvement in microbial food webs in the gastrointestinal tract and pathogenesis, amongst other roles. For this *in silico* screen, BAGEL4 was used to mine the genomes of 1,136 strains belonging to this Bacteroidales order. This revealed 1,340 areas of interest (AOI) and it was noted radical S-adenosyl methionine (rSAM) enzymes were the most commonly identified RiPP associated enzymes. These enzymes were detected either independently or in combination with other enzymes involved in biosynthesis such as YcaO. This is notable as radical SAM enzymes are a common feature involved in the biosynthesis of the sactipeptide subclass of RiPPs. The authors subsequently focused on the analysis of nine BGCs in detail and found a clear association between precursor peptides containing GG-motifs and the presence of peptidase containing ATP-binding transporters (PCATs). It was hypothesized that these PCATs are likely involved in cleavage of the leader peptide and transport of the mature products. Overall, this study highlighted the potential for Bacteroidales to produce RiPPs and, since Bacteroidales are a key component of the human microbiome, these traits have the potential to be harnessed to strategically shape the human microbiome.

Finally, one review article published as part of this RT focused on providing a comprehensive overview of probiotic streptococcal strains that produce bacteriocin like inhibitory substances (BLIS), while also discussing how these can be harnessed to beneficially modulate the microbiome in the oral cavity (Tagg et al.). The oral microbiota consists of a heterogeneous population of microbes which exist in a homeostatic nature in a healthy oral cavity. However, as is the case with other microbiomes such as that found in the gut, dysbiosis can occur as a consequence of a variety of factors, examples of which include poor nutritional status, oral infections and alterations in host physiology, amongst other such factors. Oral dysbiosis can lead to conditions such as dental caries, halitosis, periodontal disease and streptococcal sore throats. Current approaches for treating such conditions involve broad-spectrum antimicrobials, chemical approaches, physical cleaning and debridement, with the ultimate aim of removing the etiological pathogens. However, it was noted that perhaps a more targeted and narrow-spectrum approach is warranted to eliminate the target pathogen and minimize the so called “collateral damage” and disruption of homeostasis that occurs to the oral microbiome as a consequence of the above-mentioned techniques. Tagg et al. document studies and evidence relating to probiotic strains that produce BLIS. The key advantage of BLIS and BLIS-producing probiotics over more broad-spectrum antimicrobials is their potential to target key pathogens in the oral cavity and help restore microbiome homeostasis in a much more expeditious manner. BLIS K12 and BLIS M18 are two examples of BLIS produced by probiotic strains of *Streptococcus salivarius*, which are commensals of the oral cavity, and these can be harnessed to restore oral microbiome homeostasis. The review article also highlighted the discovery of other streptococcal and non-streptococcal probiotics that could have similar applications. Although the primary focus of the review article was the oral microbiome, the authors also briefly mentioned that such BLIS producing probiotics and BLIS compounds could have potential in other applications pertaining to the human microbiome in the future.

Thus, overall, this Research Topic is composed of a collection of hugely fascinating and relevant studies pertaining to bacteriocins, RiPPs, and perhaps most importantly, their applications in biotechnology, human health and strategic manipulation of different microbiome contexts. This diverse series of studies involves different classes of bacteriocins and RiPPs and explores a wide range practical applications. As a result, researchers working on bacteriocins and RiPPs, as well as a broader audience interested in microbiology, will find these studies to be interesting and informative.

## Data Availability

The original contributions presented in the study are included in the article/supplementary material, further inquiries can be directed to the corresponding author/s.

